# Enhanced Triacylglycerol Production With Genetically Modified *Trichosporon oleaginosus*

**DOI:** 10.3389/fmicb.2018.01337

**Published:** 2018-06-21

**Authors:** Kari Koivuranta, Sandra Castillo, Paula Jouhten, Laura Ruohonen, Merja Penttilä, Marilyn G. Wiebe

**Affiliations:** VTT Technical Research Centre of Finland Ltd., Espoo, Finland

**Keywords:** triacylglycerol, lipid production, *Trichosporon oleaginosus*, yeast, enhanced yield, *Cryptococcus curvatus*, pyruvate dehydrogenase bypass

## Abstract

Mitochondrial pyruvate dehydrogenase (PDH) is important in the production of lipids in oleaginous yeast, but other yeast may bypass the mitochondria (PDH bypass), converting pyruvate in the cytosol to acetaldehyde, then acetate and acetyl CoA which is further converted to lipids. Using a metabolic model based on the oleaginous yeast *Yarrowia lipolytica*, we found that introduction of this bypass to an oleaginous yeast should result in enhanced yield of triacylglycerol (TAG) on substrate. *Trichosporon oleaginosus* (formerly *Cryptococcus curvatus*) is an oleaginous yeast which can produce TAGs from both glucose and xylose. Based on the sequenced genome, it lacks at least one of the enzymes needed to complete the PDH bypass, acetaldehyde dehydrogenase (ALD), and may also be deficient in pyruvate decarboxylase and acetyl-CoA synthetase under production conditions. We introduced these genes to *T. oleaginosus* in various combinations and demonstrated that the yield of TAG on both glucose and xylose was improved, particularly at high C/N ratio. Expression of a phospholipid:diacyltransferase encoding gene in conjunction with the PDH bypass further enhanced lipid production. The yield of TAG on xylose (0.27 g/g) in the engineered strain approached the theoretical maximum yield of 0.289 g/g. Interestingly, TAG production was also enhanced compared to the control in some strains which were given only part of the bypass pathway, suggesting that these genes may contribute to alternative routes to cytoplasmic acetyl CoA. The metabolic model indicated that the improved yield of TAG on substrate in the PDH bypass was dependent on the production of NADPH by ALD. NADPH for lipid synthesis is otherwise primarily supplied by the pentose phosphate pathway (PPP). This would contribute to the greater improvement of TAG production from xylose compared to that observed from glucose when the PDH bypass was introduced, since xylose enters metabolism through the non-oxidative part of the PPP. Yield of TAG from xylose in the engineered strains (0.21–0.27 g/g) was comparable to that obtained from glucose and the highest so far reported for lipid or TAG production from xylose.

## Introduction

Biofuels such as biodiesel continue to be favorites as renewable transportation fuels, since their use requires little modification to current engines. Biodiesel and renewable diesel can be produced from renewable biological sources such as vegetable oils and animal fats. They are biodegradable, non-toxic, and have a low emission profile. Due to the increased demand for biodiesel and the limited and/or undesirable sources of biodiesel raw materials such as rape seed oil, soy bean oil, or palm oil, it is of importance to expand biodiesel raw materials to non-food materials like microbes. The benefits of using microbes for production of oils are that they are affected neither by seasons nor by climates, they are able to produce high lipid contents, and the oils can be produced from a wide variety of sources with short production times (Adrio, 2017). Here we show how triacylglycerol (TAG) yield in the oleaginous yeast *Trichosporon oleaginosus* (formerly *Cryptococcus curvatus*) can be improved by modifying central metabolic pathways.

**FIGURE 1:**
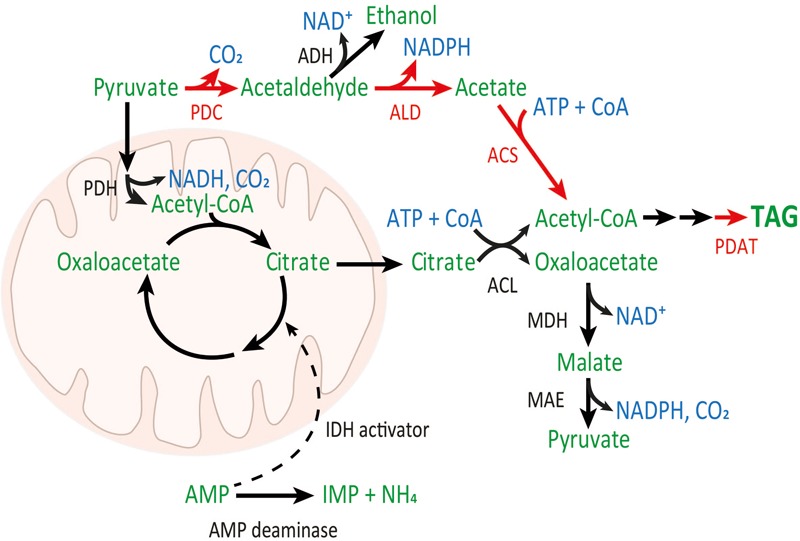
PDH (black arrows) and PDH bypass (red arrows) pathways to cytosolic acetyl-CoA. PDH and TCA reactions occur within the mitochondria. Relevant enzymes: pyruvate dehydrogenase (PDH), pyruvate decarboxylase (PDC), alcohol dehydrogenase (ADH), acetaldehyde dehydrogenase (ALD), acetyl-CoA synthetase (ACS), ATP:citrate lyase (ACL), phospholipid:diacylglycerol acyltransferase (PDAT), malate dehydrogenase (MDH), and malic enzyme (MAE). Metabolites are shown in green, and cofactors and CO_2_ are shown in blue. Genes which were introduced to *T. oleaginosus* are indicated in red.

A few fungal species accumulate remarkable amounts of lipid in the cells, the majority of which is TAG. It has been observed that lipids accumulate in these oleaginous fungi under nitrogen limited conditions, leading to the following hypothesis as to why lipid accumulation occurs (Ratledge and Wynn, 2002). Nitrogen limitation causes activation of an adenosine monophosphate (AMP) deaminase, which utilizes AMP to produce NH_4_. The decrease in AMP concentration inhibits the activity of mitochondrial isocitrate dehydrogenase (IDH), which is part of the mitochondrial tricarboxylic acid (TCA) cycle. Decrease in IDH activity results in citrate accumulation. Excess citrate is transferred to the cytosol where ATP:citrate lyase (ACL) activity converts it and coenzyme A (CoA) to acetyl-CoA and oxaloacetate, with ATP hydrolysis (**Figure 1**).

Cytosolic acetyl-CoA can be used in fatty acid synthesis (Tehlivets et al., 1997), resulting in palmitoyl-acyl carrier protein (ACP; C16:0). Palmitic acid and other intermediates of fatty acid synthesis, after hydrolysis to acyl-CoAs by a hydrolase or thioesterase, can be further modified by various elongases and desaturases to acyl-chains of different lengths, with or without double bonds. In one cycle of fatty acid synthesis, two NADPHs are required in the reduction steps. These acyl-CoAs may also be incorporated to TAGs. In the last step of TAG synthesis, diacylglycerol is acylated to TAG, mainly by acyl-CoA:diacylglycerol acyltransferase (DGAT) and phospholipid:diacylglycerol acyltransferase (PDAT), utilizing acyl-CoA or phosphatidylcholine, respectively, as acyl donors (Sorger and Daum, 2003).

In oleaginous microbes, the lipid precursor, cytosolic acetyl-CoA, is produced via the above-mentioned mitochondrial pyruvate dehydrogenase (PDH) pathway (**Figure 1**). However, in non-oleaginous yeast like *Saccharomyces cerevisiae*, cytosolic acetyl-CoA is produced via the PDH bypass (**Figure 1**). In the PDH bypass, the enzymes pyruvate decarboxylase (PDC), acetaldehyde dehydrogenase (ALD), and acetyl-CoA synthetase (ACS) have essential roles in cytosolic acetyl-CoA and lipid production (**Table 1**), as well as in the generation of reducing equivalents (NADH and NADPH; Flikweert et al., 1996; Pronk et al., 1996; Saint-Prix et al., 2004). The regulation and attainable lipid yield of these two pathways differ, particularly in the response to nitrogen limitation (which is important in initiating acetyl-CoA accumulation in the mitochondrial path, but not in the PDH bypass) and in the generation of reducing equivalents (van Rossum et al., 2016; Xu et al., 2016). A comparison of the stoichiometry of these pathways in *S. cerevisiae* showed that the attainable lipid yield through the PDH bypass is higher than that delivered through the PDH pathway (van Rossum et al., 2016). Thus, introducing a PDH bypass to an oleaginous yeast lacking this pathway could improve the yield of lipid from carbohydrate.

**Table 1:** Genes overexpressed in this study.

Gene	Gene name and database ID	Source of gene	Function
*PDC1*	Pyruvate decarboxylase (SGD:S000004034)	*Saccharomyces cerevisiae*	Catalyzes decarboxylation of pyruvate to acetaldehyde and carbon dioxide
*ALD6*	Acetaldehyde dehydrogenase (SGD:S000005982)	*Saccharomyces cerevisiae*	Catalyzes the oxidation of acetaldehyde to acetate, with the reduction of NADP^+^ to NADPH
*ACS2*	Acetyl-CoA synthetase (SGD:S000004143)	*Saccharomyces cerevisiae*	Catalyzes the formation of acetyl-CoA acetate and CoA, with hydrolysis of ATP
*PDAT*	Phospholipid:diacylglycerol acyltransferase (GenBank:RO3G_07851)	*Rhizopus oryzae*	Catalyzes the transfer of an acyl group from a phospholipid to 1,2-diacylglycerol at position sn-3

Here we demonstrate that introducing the PDH bypass route for producing cytosolic acetyl-CoA to the oleaginous yeast *T. oleaginosus* resulted in an increase in the yield of TAG on glucose or xylose, as also predicted in metabolic model simulations. We also showed that expression of a PDAT encoding gene in conjunction with the PDH bypass further enhanced lipid production. With these modifications, we enhanced TAG yield on both biomass and on carbohydrate by about 30% compared to the control, particularly when xylose was provided as substrate.

## Materials and Methods

### Strains, Media, and Growth Conditions

*Escherichia coli* Top10 (Thermo Fisher Scientific Inc., Waltham, MA, United States) was used for all cloning purposes throughout this study and maintained on LB medium with ampicillin (100 μg/ml) at 37°C. *T. oleaginosus* (formerly *C. curvatus*) ATCC20509 was used as a host for genetic engineering. Genes required for the PDH bypass and PDAT, as described in **Table 1**, were introduced individually and in combination with other genes for the bypass to generate the strains described in **Table 2**. Genes (*PDC1, ACS2*, and *ALD6*) were added singly as well as in combination in order to assess whether the PDH bypass genes could have effects independent of the complete pathway. Data for strains with single gene additions are presented in Supplementary Tables S1–S3. All *T. oleaginosus* strains were maintained on yeast extract (10 g/l), peptone (20 g/l) plus glucose (YPD) or xylose (YPX) with agar (20 g/l) at 30°C.

**Table 2:** Plasmids and *T. oleaginosus* strains generated.

Strain	Plasmids	Genotype
*PDC1*	pKK101	TEF1_p_-G418-TPI1_t_-TPI_p_-ScPDC1-TEF1_t_
*ALD6*	pKK81	TEF1_p_-Hph-TPI1_t_-ENO1_p_-ScALD6-TEF1_t_
*ACS2*	pKK86	TEF1_p_-Hph-TPI1_t_-TPI1_p_-ScACS2-ENO1_t_
*PDAT*	pKK95	TEF1_p_-CerR-TPI1_t_-TPI1_p_-RoPDAT-GPD1_t_
*ACS2, ALD6*	pKK85	TEF1_p_-Hph-TPI1_t_-TPI1_p_-ScACS2-ENO1_t_-ENO1p-ScALD6-TEF1_t_
*PDC1, ALD6*	pKK101+pKK81	TEF1_p_-Hph-TPI1_t_-ENO1_p_-ScALD6-TEF1_t_- TEF1_p_-G418-TPI1_t_-TPI_p_-ScPDC1-TEF1_t_
*PDC1, ACS2*	pKK101+pKK86	TEF1_p_-Hph-TPI1_t_-TPI1_p_-ScACS2-ENO1_t_- TEF1_p_-G418-TPI1_t_-TPI_p_-ScPDC1-TEF1_t_
*PDC1, PDAT*	pKK101+pKK95	TEF1_p_-CerR-TPI1_t_-TPI1_p_-RoPDAT-GPD1_t_- TEF1_p_-G418-TPI1_t_-TPI_p_-ScPDC1-TEF1_t_
*ALD6, PDAT*	pKK81+pKK95	TEF1_p_-Hph-TPI1_t_-ENO1_p_-ScALD6-TEF1_t_- TEF1_p_-CerR-TPI1_t_-TPI1_p_-RoPDAT-GPD1_t_
*PDC1, ALD6, ACS2*	pKK101+pKK85	TEF1_p_-G418-TPI1_t_-TPI_p_-ScPDC1-TEF1_t_: TEF1_p_-Hph-TPI1_t_-TPI1_p_-ScACS2-ENO1_t_-ENO1p-ScALD6-TEF1_t_
*PDC1, ALD6, PDAT*	pKK101+pKK81+pKK95	TEF1_p_-G418-TPI1_t_-TPI_p_-ScPDC1-TEF1_t_: TEF1_p_-Hph-TPI1_t_-ENO1_p_-ScALD6-TEF1_t_- TEF1_p_-CerR-TPI1_t_-TPI1_p_-RoPDAT-GPD1_t_
*ALD6, ACS2, PDAT*	pKK85+pKK95	TEF1_p_-Hph-TPI1_t_-TPI1_p_-ScACS2-ENO1_t_-ENO1p-ScALD6-TEF1_t_-TEF1_p_-CerR-TPI1_t_-TPI1_p_-RoPDAT-GPD1_t_
*PDC1, ALD6, ACS2, PDAT*	pKK101+pKK85+pKK95	TEF1_p_-G418-TPI1_t_-TPI_p_-ScPDC1-TEF1_t_: TEF1_p_-Hph-TPI1_t_-TPI1_p_-ScACS2-ENO1_t_-ENO1p-ScALD6-TEF1_t_-TEF1_p_-CerR-TPI1_t_-TPI1_p_-RoPDAT-GPD1_t_

Cultivation medium (pH 5.5) in flask cultivations with initial C/N ratio 20 contained 20 g xylose, 0.3 g (NH_4_)_2_SO_4_, 7.0 g KH_2_PO_4_, 2.5 g Na_2_HPO_4_⋅H_2_O, 1.5 g MgSO_4_⋅7H_2_O, 4.0 g yeast extract, 51 mg CaCl_2_, 8 mg FeCl_3_⋅6 H_2_O, and 0.1 mg ZnSO_4_⋅H_2_O per liter. The same concentrations of yeast extract and mineral salts with 30 g/l glucose resulted in a C/N ratio of 28. Cultivation medium with initial C/N ratio 65 was similar to the C/N 20 medium except that the concentration of yeast extract was reduced to 0.6 g/l with 20 g/l glucose or xylose. The cultivation medium with C/N ratio 103 contained 0.15 g (NH_4_)_2_SO_4_ and 0.45 g yeast extract per liter as nitrogen source, with 20 g/l glucose or xylose. In some cultures, the glucose concentration was increased to 30 g/l, resulting in an initial C/N ratio of 153. Each flask (50 ml medium in 250 ml flasks) was inoculated to an OD_600_ of 0.3 with cells grown on YPD or YPX plates. The cultivations were maintained at a temperature of 30°C with shaking at 250 rpm. Samples were analyzed for cell dry weight (CDW), lipid and TAG content, and residual carbohydrate (by HPLC).

In bioreactor cultivations, transformants and wild type *T. oleaginosus* were cultivated in Multifors bioreactors (max. working volume 500 ml, Infors HT, Switzerland) at pH 4.0, 30°C, in 500 ml medium (initial C/N ratio 70) containing 94 ± 0.4 g glucose, 2.56 g (NH_4_)_2_SO_4_, 1.2 g KH_2_PO_4_, 0.3 g Na_2_HPO_4_ ⋅2H_2_O, 1.5 g MgSO_4_⋅7H_2_O, 0.1 g CaCl_2_⋅6H_2_O, 5.26 mg citric acid⋅H_2_O, 5.26 mg ZnSO_4_⋅7H_2_O, 0.1 mg MnSO_4_⋅4H_2_O, 0.5 mg CoCl_2_⋅6H_2_O, 0.26 mg CuSO_4_⋅5H_2_O, 0.1 mg Na_2_MoO_4_⋅2H_2_O, 1.4 mg FeSO_4_⋅7H_2_O, 0.1 mg H_3_BO_4_, 0.05 mg D-biotin, 1.0 mg Ca pantothenate, 5.0 mg nicotinic acid, 25 mg myoinositol, 1.0 mg thiamine⋅HCl, 1.0 mg pyridoxine⋅HCl, and 0.2 mg *p*-aminobenzoic acid per liter. The pH was maintained constant by addition of 1 M KOH or 1 M H_2_PO_4_. Cultures were agitated at 1000 rpm (two Rushton turbine impellors) and aerated at 2 volumes air per volume culture per minute (vvm). Clerol FBA 3107 antifoaming agent (Cognis, Saint-Fargeau-Ponthierry Cedex France, 1 ml/l) was added to prevent foam accumulation. Bioreactors were inoculated to initial OD_600_ of 0.5–4.0 with cells grown in the same medium [substituting 1.5 g urea per liter for (NH_4_)_2_SO_4_ and omitting the CaCl_2_⋅6H_2_O] in 50 ml volumes in 250 ml flasks at 30°C with shaking at 200 rpm for 24–42 h. Samples for CDW measurement, lipid extraction, and HPLC analysis were withdrawn periodically during cultivation. CDW was determined by centrifuging 0.5–2.0 ml culture broth in pre-dried, pre-weighed 2 ml microfuge tubes. After washing twice with 1.8 ml distilled water, the cell pellet was dried at 100°C for 48 h and weighed after cooling in a desiccator.

In the cultivation with xylose, the medium contained 94 ± 0.6 g xylose per liter, instead of glucose. The xylose cultivations were inoculated to initial OD_600_ of 17–24 with cells grown in low nitrogen medium with glucose as carbon source in the Multifors bioreactors at 30°C.

### HPLC Analytics

HPLC analyses for sugars were conducted with a Waters 2690 Separation Module and Water System Interfase Module liquid chromatography coupled with a Waters 2414 differential refractometer and Waters 2487 dual absorbance detector. The liquid chromatography columns were a 100 mm × 7.8 mm Fast Acid Analysis column from Bio-Rad and a 300 mm × 7.8 mm Aminex HPX-87H column from Bio-Rad. The columns were equilibrated with 2.5 mM H_2_SO_4_ in water at 55°C and samples were eluted with 2.5 mM H_2_SO_4_ in water at 0.5 ml/min flow rate. Data acquisition was done using Waters Millennium software.

### Gene Synthesis

*Saccharomyces cerevisiae PDC1, ALD6, ACS2*, and *Rhizopus oryzae PDAT* genes (see descriptions in **Table 1**) were codon optimized according to *Ustilago maydis* yeast codon usage and synthetized by Thermo Fisher Scientific Inc. (Waltham, MA, United States).

The *E. coli* hygromycin (hph) gene, that confers resistance to hygromycin B, was polymerase chain reaction (PCR) amplified from plasmid pRLMEX30 (Mach et al., 1994) DNA. The *E. coli* G418 resistance gene was PCR amplified using plasmid pPIC9K (Invitrogen, Thermo Fisher Scientific Inc., Waltham, MA, United States) DNA as template. The *S. cerevisiae* cerulenin resistance gene was PCR amplified using the cerulenin resistance gene from plasmid pCR1 (Nakazawa et al., 1993) as template.

### Plasmid Constructions

Polymerase chain reactions for cloning purposes were performed with Dynazyme EXT DNA polymerase (Thermo Fisher Scientific Inc., Waltham, MA, United States) according to the manufacturer’s instructions. All primers used are listed in Supplementary Table S4. PCR products were cloned into pBluescript KS plasmid [Agilent (Stratagene), Santa Clara, CA, United States] and after verification of the PCR products by sequencing, they were released for subsequent cloning purposes by digestion with suitable restriction endonucleases.

Genomic fragments containing *T. oleaginosus* promoter and terminator regions were obtained by ligation-mediated PCR amplification (Mueller and Wold, 1989). A mixture of a PCR linker I and a PCR linker II was ligated to *Pvu*II, *Ssp*I, or *Nru*I digested *T. oleaginosus* genomic DNA with T4 DNA ligase (New England BioLabs). Samples of the ligation mixtures were used as templates in the first PCR reaction, which contained a PCR linker I primer and a gene-specific primer. A diluted sample of this first PCR amplification was used as the template in a nested PCR reaction containing a PCR Linker I primer and gene-specific nested primer. Yeast gene-specific primers were degenerative primers designed from a consensus sequence of the putative genes of *Ustilago maydis, Candida guilliermondii*, and *Candida tropicalis.*

### Yeast Transformation

Plasmids were digested to release integration cassettes containing marker gene and gene(s) to be expressed under endogenous promoters and terminators as indicated in **Table 2**. *T. oleaginosus* was cultivated overnight in 50 ml of YPD with 250 rpm shaking at 30°C. When the culture OD_600_ reached 20, cells were centrifuged at 3200 *g* for 4 min and the pellet was washed once with 5 ml of ice-cold EB buffer (10 mM Tris-HCl, pH 7.5, 270 mM sucrose, 1 mM MgCl_2_). The pellet was re-suspended in 5 ml of IB buffer (25 mM DTT, 20 mM Hepes, pH 8.0 in YPD) and incubated at 30°C for 30 min with 250 rpm shaking. After incubation, cells were harvested by centrifugation (3200 *g*, 5 min) and washed once with 5 ml of EB buffer and re-suspended in 500 μl of EB buffer; 400 μl of cell suspension was transferred into a 0.4 cm electroporation cuvette with 6 μg of DNA in 50 μl of water or 10 mM Tris-HCl, pH 7.5. After samples had been 15 min on ice, electroporation was carried out with a Bio-Rad (Hercules, CA, United States) Gene Pulser (1800 V, 1000 Ω, 25 μF). After electroporation, 1 ml YPD was added and samples were incubated in 50 ml falcon tubes for 4 h at 30°C with 250 rpm shaking; 50–200 μl of suspension was plated on YPD-agar plates containing G418 (200 μg/ml), hygromycin (100 μg/ml), or cerulenin (2 μg/ml). Transformants were confirmed by PCR.

### Lipid Extraction and Total Lipid and Triglyceride Concentration Measurements

The lipid extraction method was modified from the protocol of Folch et al. (1957). Yeast cell culture (0.5–2 ml) was centrifuged in a microcentrifuge tube (13,000 rpm, 1 min) and the supernatant discarded. The pellet was placed rapidly in liquid nitrogen and stored at -80°C. The frozen pellet was suspended in 500 μl of ice-cold methanol with 0.1% BHT (2,6-di-*tert*-butyl-4-methylphenol) and homogenized with a Mixer Mill homogenizer with 5-mm zirconium oxide and 3-mm yttrium stabilized zirconium oxide balls (Retsch GmbH, Germany) at 25 Hz for 5 min. After homogenizing in methanol, 1000 μl of chloroform was added and the homogenization repeated, then 300 μl of 20 mM acetic acid was added, and the sample vortexed for 10 min. The vortexed sample was centrifuged at 13,000 rpm for 5 min at room temperature. The lower phase was recovered and 1000 μl of chloroform was added to the remaining phase, vortexed, and re-centrifuged. The lower phases were combined into pre-weighed 2 ml microfuge tubes, and dried, after which the total lipid content of the sample was determined by gravimetry. Then the lipid sample was re-dissolved in 1.5 ml of chloroform:methanol (2:1) + 0.1% BHT and stored at -20°C.

For TAG analysis, 100–1500 μl of chloroform:methanol extracted lipids was evaporated and re-dissolved in 200–1000 μl of isopropanol. TAGs were measured enzymatically from the samples using the Konelab Triglycerides Kit (Thermo Fisher Scientific Inc., Waltham, MA, United States) and a Cobas Mira automated analyzer (Roche, Basel, Switzerland) or a microtiter-plate reader (Varioskan, Thermo Fisher Scientific Inc., Waltham, MA, United States).

### Statistical Analysis

Results are given as mean ± standard error of the mean (SEM). Comparisons were made by analysis of variance (ANOVA), followed by Fisher’s multiple range test when the ANOVA indicated significant differences. For direct comparison with the control strain, a Student’s *t*-test was also used, as indicated in the text. *P*-values less than 0.10 but greater than 0.05 were considered to show slight differences between strains, although not significant at *p* < 0.05.

### Metabolic Model Simulations

The *Yarrowia lipolytica* genome scale metabolic model^1^ was used to represent the metabolism of an oleaginous yeast which uses the PDH route with ATP citrate lyase for lipid production, as no curated metabolic model was available for *T. oleaginosus*. A full PDH bypass was introduced to the model to simulate the maximum theoretical TAG yield through the different pathway options. An NADP^+^ dependent version of ALD was also introduced. No simultaneous forward and reverse IDH flux was allowed through NADP^+^ and NAD^+^ dependent isoforms. Flux variability analysis (Burgard et al., 2001; Mahadevan and Schilling, 2003) was performed with COBRApy^2^ with glpk v. 4.63 LP solver.

The conversion of molar yields to g/g was done by assuming the FA composition of acyl-CoA in the model^1^, giving a molar mass of 826.678 g/mol for TAG.

## Results

### Model Simulation for the PDH Route and PDH Bypass

Maximum attainable TAG yields through the PDH and the PDH bypass routes were estimated from genome-scale metabolic model simulations. In the model simulation, a maximum limit for flux to the oxidative pentose phosphate pathway (PPP) was set to 52.0% of glucose uptake, based on ^13^C flux analysis of *Y. lipolytica* wild type strain MTYL037 (Wasylenko et al., 2015). The PPP is the major source of NADPH in cells, for, e.g., FA synthesis, and is also carbon-efficient. With the amount of NADPH provided through the PPP limited, the metabolic stoichiometry would allow glucose conversion to TAG through the PDH and PDH bypass routes with yields 0.275 and 0.289 g/g, respectively. However, TAG synthesis from glucose through the PDH route relied on metabolite cycling in mitochondria, with an unrealistically high flux through the malic enzyme. Therefore, a constraint for the maximum flux through the malic enzyme of 41.5% of glucose uptake was introduced, based on experimental evidence (Wasylenko et al., 2015). With this upper bound for malic enzyme flux, the estimated attainable yields of TAG from glucose through the PDH and PDH bypass routes were 0.252 and 0.289 g/g (unchanged), respectively. Thus, the genome-scale metabolic model simulations estimated that the PDH bypass route would be more favorable for TAG synthesis in an oleaginous yeast than the PDH route. The higher yield was dependent on the ALD reaction in the PDH bypass, in which NADPH is generated from NADP^+^.

### Flask Cultivations on Glucose With C/N Ratio 65

**FIGURE 2:**
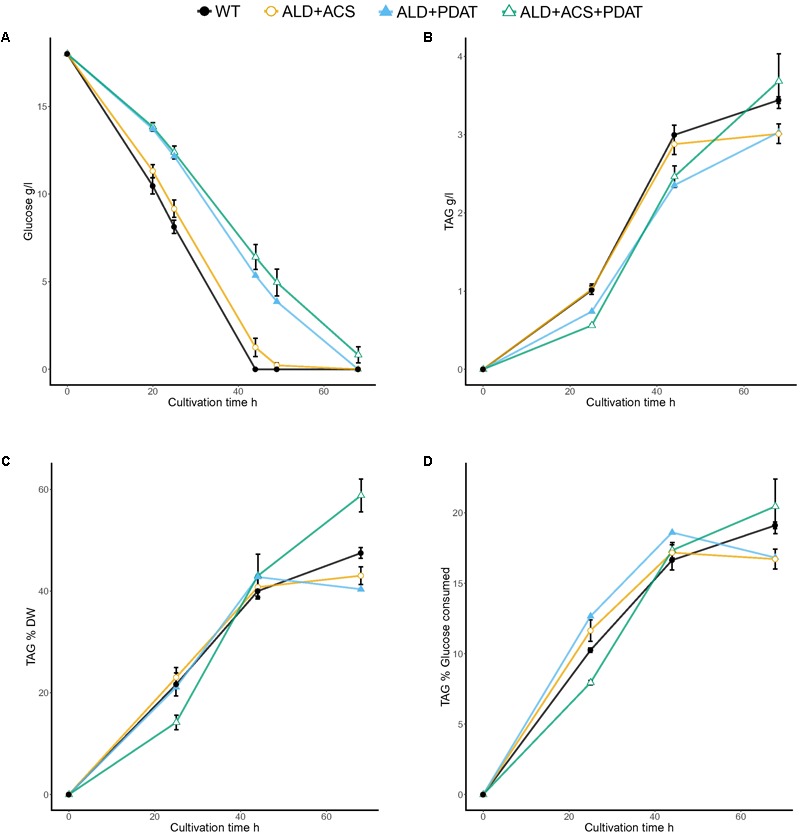
Glucose consumption **(A)** and TAG production [titer **(B)** and yields *Y*_TAG/x_ **(C)** and *Y*_TAG/glucose_ **(D)**] from glucose at C/N ratio 65 in flask cultivation. Error bars present ± SEM and if not visible are smaller than the symbol.

Transformants having *S. cerevisiae ALD6* (5), *S. cerevisiae ACS2* (5) or both *S. cerevisiae ALD6* and *ACS2* (5), *R. oryzae PDAT* (2), or *S. cerevisiae ALD6* and *ACS2* and *R. oryzae PDAT* (2), and one transformant having *S. cerevisiae ALD6* and *R. oryzae PDAT* were cultivated in flasks with 20 g/l glucose at C/N ratio 65 (**Figure 2** and Supplementary Table S1). Individual transformants (the number of which is indicated in parentheses above) were used as replicates when assessing the ability of the new strains to produce TAG from glucose. Since the strains were generated by random integration, independent transformants may differ in the efficiency at which the added genes were expressed, increasing the variation between replicates. Wild type *T. oleaginosus* was cultivated as a control (**Figure 2**).

The control strain and transformants having *ALD6* and/or *ACS2* genes consumed glucose faster than the transformants which had *PDAT*. The ACS2 strains, for which there were several transformants, produced 3.6 ± 0.07 g/l TAG, which was significantly (*p* < 0.05, *t*-test) more than produced by the control (3.4 ± 0.04 g/l). All other transformants produced similar or less TAG than the parent (**Figure 2**). The TAG yield per biomass (*Y*_TAG/x_) at the end of the cultivation was highest (*p* < 0.05) in transformants having *ALD6, ACS2*, and *PDAT* genes (58.8 ± 3.2% TAG, **Figure 2**) and in transformants with *ACS2* (52.6 ± 1.5%; Supplementary Table S1). The control strain produced only 47.5 ± 1.1% *Y*_TAG/x_ and other transformants less (**Figure 2**). Strains having *ALD6, ACS2*, and *PDAT* achieved 20.5 ± 1.9% yield per gram glucose consumed (*Y*_TAG/S_). The *Y*_TAG/S_ for the *ACS2* transformants (20.1 ± 0.37%) was significantly higher (*p* < 0.05, *t*-test) than the control strain (19.1 ± 0.2%), suggesting that more replicates would have shown the benefit in the *ALD6, ACS2*, and *PDAT* transformants also.

### Flask Cultivations on Glucose at C/N Ratio 103

**FIGURE 3:**
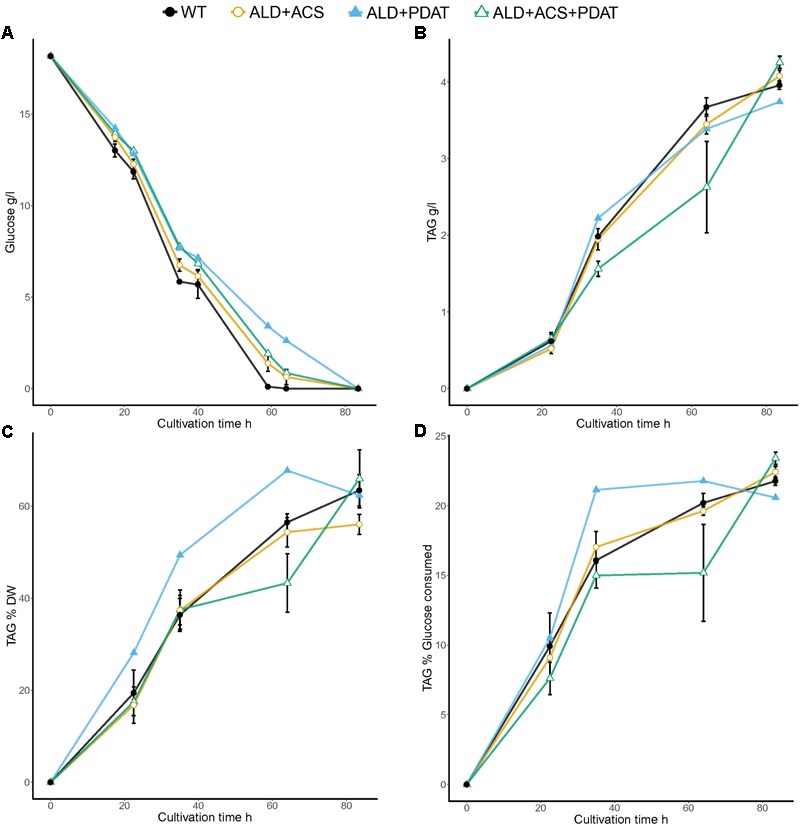
Glucose consumption **(A)** and TAG production [titer **(B)** and yields *Y*_TAG/x_ **(C)** and *Y*_TAG/glucose_ **(D)**] from glucose at C/N ratio 103 in flask cultivation. Error bars present ± SEM and if not visible are smaller than the symbol.

In the flask cultivation on glucose at C/N ratio 103, the control strain and the transformants which had *ACS2* alone consumed glucose faster than the other strains: after 59 h, there was less than 0.2 g/l residual glucose for these strains and all glucose had been consumed after 64 h, whereas the other strains still had 0.2–2.6 g/l residual glucose at 64 h (**Figure 3** and Supplementary Table S2).

All strains produced more TAG at higher yields (*Y*_TAG/x_ and *Y*_TAG/S_) at C/N ratio 103 than at C/N ratio 65 on glucose. The TAG production was highest with the strains having *ALD6, ACS2*, and *PDAT* (4.3 ± 0.1 g/l), as in the cultivation at C/N ratio 65, which was slightly higher (*p* < 0.10) than TAG produced by the control strain (4.0 ± 0.0 g/l TAG). The *PDAT* transformants also produced slightly more TAG (4.2 ± 0.2 g/l; *p* < 0.10, Supplementary Table S2) than the control, while other transformants produced around 4.0 g/l TAG, like the control (**Figure 3** and Supplementary Table S2).

At the end of the cultivation (84 h), the highest TAG yield per biomass was detected in transformants having *PDAT* (67.6 ± 5.3%), although this did not differ significantly (*p* > 0.10) from the *Y*_TAG/x_ of other transformants (56.1–66.0%) or the control strain (63.5 ± 3.4%). At C/N 103, the variation in TAG accumulation between transformants with the same genotype was more variable than was observed at C/N 65. The yield of TAG on glucose was highest (*p* < 0.10) with the transformants which had all three genes, *ALD6, ACS2*, and *PDAT* (23.4 ± 0.5%), as at C/N 65. The transformants with only *PDAT* added also produced significantly more (*p* < 0.05, *t*-test) TAG per gram glucose (23.0 ± 0.5%) than the control strain (21.8 ± 0.3%), whereas other transformants did not (*p* > 0.10, Supplementary Table S2).

### Flask Cultivations on Xylose at C/N Ratio 103

**FIGURE 4:**
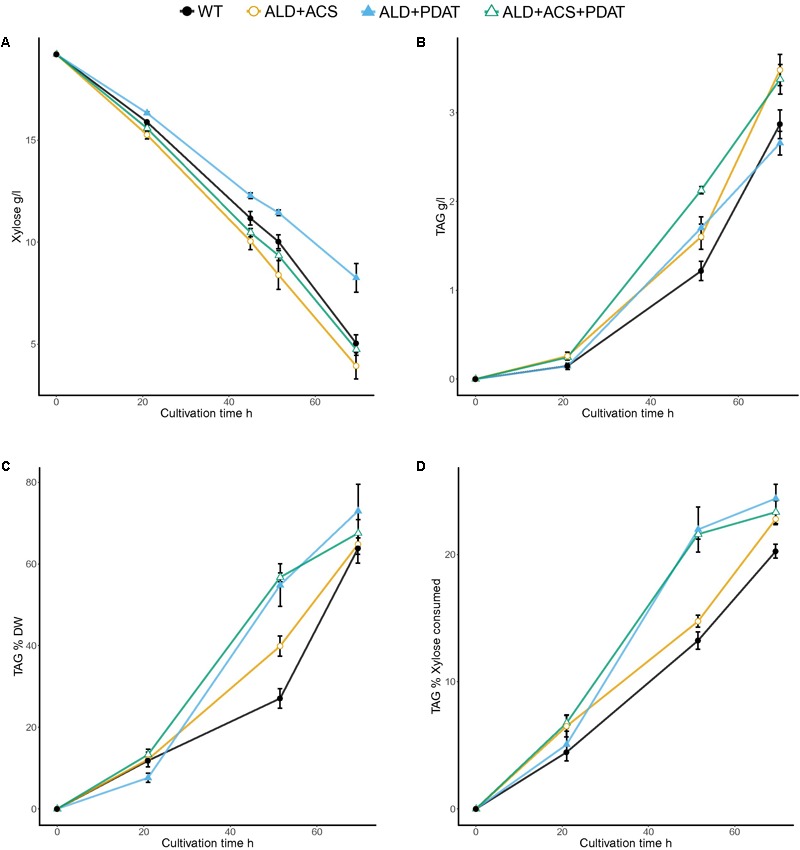
Xylose consumption **(A)** and TAG production [titer **(B)** and yields *Y*_TAG/x_ **(C)** and *Y*_TAG/xylose_ **(D)**] from xylose at C/N ratio 103 in flask cultivation. Error bars present ± SEM and if not visible are smaller than the symbol.

In the flask cultivation with xylose as carbon source and C/N ratio 103, no strain had consumed all of the xylose within 70 h, when the cultivations were stopped (**Figure 4** and Supplementary Table S3). Xylose consumption rates (0.16–0.24 g/l/h) were lower than glucose consumption rates (0.24–0.28 g/l/h at 64 h). The transformants with *ACS2* had the lowest amount (2.7 ± 0.5 g/l) and the *ALD6* and *PDAT* transformants had the highest amount of residual xylose (8.3 ± 0.7 g/l) at the end of the cultivation (**Figure 4** and Supplementary Table S3). The highest (*p* < 0.05) TAG titer was achieved by the transformants having *ALD6* (4.0 ± 0.1 g/l TAG) or *ACS2* (3.9 ± 0.1 g/l). All transformants, except those with *ALD6* and *PDAT* (2.7 ± 0.1 g/l) and *ALD6, ACS2*, and *PDAT* (3.4 ± 0.2 g/l, *p* < 0.06, *t*-test) produced significantly (*p* < 0.05) more TAG (3.5–4.0 g/l) than the control strain (2.9 ± 0.2 g/l TAG). *Y*_TAG/x_ and *Y*_TAG/S_ were highest in the transformants with the *ALD6* gene (75.9 ± 1.1 and 24.9 ± 0.6%, respectively, Supplementary Table S3). All other transformants, except those with *ALD6* and *ACS2*, also produced significantly higher (*p* < 0.05) *Y*_TAG/S_ on xylose than the control (20.3 ± 0.6%). The yield on biomass was highly variable and only significantly higher than the control (63.8 ± 3.6%) at *p* < 0.10 for the *ALD6* transformant (Supplementary Table S3).

### Flask Cultivation on Glucose With Transformants Having the *PDC1* Gene

Transformants having the *S. cerevisiae PDC1* gene expressed in different combinations with the *S. cerevisiae ALD6, ACS2*, and *R. oryzae PDAT* genes were cultivated in flasks with 30 g/l glucose and a C/N ratio of 28 or 153 (**Table 3**). TAG production was assessed after 94 h cultivation, at which time all glucose was expected to have been consumed (**Figure 3**). However, even at C/N ratio 28, all strains still had residual glucose (2.4–6.1 g/l). Variability between replicates was high and the TAG titer (4.7–5.4 g/l) was similar (*p* > 0.10) in all strains, transformants, and control. The highest yield on biomass was achieved with the strain having only *PDC1* (60.7 ± 12.4%), whereas *Y*_TAG/x_ was lowest for the control strain (50.2 ± 6.5). The yield on glucose varied between 18.8 and 21.6% and was also highly variable (**Table 3**).

**Table 3:** Residual glucose, TAG titer (g/l), and yields (on biomass, *Y*_TAG/x_, and on glucose, *Y*_TAG/glucose_) after 94 h cultivation on glucose (30 g/l).

	C/N 28				C/N 153			
Strains	Residual glucose (g/l)	TAG (g/l)	*Y*_TAG/x_ (%)	*Y*_TAG/glucose_ (%)	Residual glucose (g/l)	TAG (g/l)	*Y*_TAG/x_ (%)	Y_TAG/glucose_ (%)
WT	3.6 ± 0.1	4.9 ± 0.5	50.2 ± 6.5	19.7 ± 1.8	5.7 ± 0.1	3.1 ± 1.3	33.8 ± 13.6	13.5 ± 5.5
*PDC1*	4.2 ± 0.3	5.1 ± 0.5	60.7 ± 12.4	21.1 ± 2.3	7.1 ± 0.4	4.2 ± 0.1	49.0 ± 3.7	19.3 ± 0.9^∗^
*PDC1, ALD6*	5.0 ± 5.8	4.7 ± 0.7	57.6 ± 14.2	20.3 ± 1.2	11.2 ± 0.7	3.2 ± 0.3	47.0 ± 12.4	17.9 ± 0.9^∗^
*PDC1, ACS2*	4.8 ± 2.8	4.8 ± 0.3	52.1 ± 2.7	20.4 ± 1.2	8.4 ± 4.0	3.9 ± 0.9	50.4 ± 7.4	18.9 ± 2.1^∗^
*PDC1, PDAT*	6.1 ± 4.0	4.7 ± 0.6	59.7 ± 4.1	21.3 ± 1.3	9.0 ± 3.5	3.7 ± 0.3	50.2 ± 3.8	18.7 ± 1.9^∗^
*PDC1, ALD6, ACS2*	3.5 ± 1.3	5.4 ± 0.1	59.8 ± 9.7	21.6 ± 1.1	6.1 ± 0.5	4.5 ± 0.6	50.0 ± 7.1	19.6 ± 2.7^∗^
*PDC1, ALD6, PDAT*	4.8 ± 0.8	4.9 ± 0.3	55.7 ± 7.3	21.1 ± 2.2	11.1 ± 0.7	4.2 ± 0.3	62.6 ± 0.9^∗^	23.3 ± 1.0^∗^
*PDC1, ALD6, ACS2, PDAT*	2.4 ± 0.8	4.9 ± 0.2	52.3 ± 2.0	18.8 ± 0.1	7.8 ± 0.2	4.5 ± 0.1	55.0 ± 0.8	21.2 ± 0.4^∗^

*Values which are significantly higher (*p* < 0.10) than the control are indicated with ^∗^.*

At C/N ratio 153, there was 5.7–11.2 g/l glucose remaining when the TAG was analyzed at 94 h (**Table 3**) and the effective C/N was between 98 and 123. The TAG production was highest with the *PDC1, ALD6, ACS2* and *PDC1, ALD6, ACS2, PDAT* transformants (4.5 g/l), but because of the variability between replicates, this did not appear to be more TAG than the control (3.14 ± 1.3, **Table 3**). TAG titers were comparable to those observed at C/N103 when strains lacking the *S. cerevisiae PDC1* gene were grown in 20 g/l glucose (**Figure 3**), but lower than expected from the growth in C/N 28 (**Table 3**). The highest *Y*_TAG/x_ was achieved with the *PDC1, ALD6, PDAT* strain (62.6 ± 0.9%) which was slightly higher (*p* < 0.10) than with the control strain (33.8 ± 13.6, **Table 3**). The *Y*_TAG/S_ was also highest (*p* < 0.10) in the *PDC1, ALD6, PDAT* strain (23.3 ± 1.0%), but other transformants also showed higher yield on glucose than the control (**Table 3**).

### Flask Cultivation on Xylose With Transformants With the *PDC1* Gene

Transformants having the *S. cerevisiae PDC1* gene expressed in different combinations with the *S. cerevisiae ALD6, ACS2* and *R. oryzae PDAT* genes were cultivated in flasks with 20 g/l xylose at C/N ratio 20 or 103 (**Table 4**). In contrast to the growth on glucose, after 94 h at C/N ratio 20, all xylose had been consumed by all strains. The control strain, *PDC1* and *PDC1, ALD6, ACS2* strains had consumed the xylose already within 70 h (data not shown). TAG production after 94 h was similar (*p* > 0.10) for all strains, including the control: 3.7–4.3 g/l TAG was produced (**Table 4**). The highest yield on biomass was detected with the strain expressing *PDC1* (71.4 ± 6.2%, **Table 4**), as in the cultivation with glucose (**Table 3**). The other strains, including the control strain, had 58.5–66.8% *Y*_TAG/x_, with the transformant with *PDC1* and *PDAT* containing less (*p* < 0.10) TAG per gram biomass than the control and most productive strains. TAG yield on xylose was highest in the *PDC1* strain (20.4 ± 0.9), but there were no significant differences between any strains (18.3–21.5%, **Table 4**).

**Table 4:** Residual xylose, TAG titer (g/l), and yields (on biomass, *Y*_TAG/x_, and on xylose, *Y*_TAG/xylose_) after 94 h cultivation on xylose (20 g/l).

	C/N 20				C/N 103			
Strains	Residual xylose (g/l)	TAG (g/l)	*Y*_TAG/x_ (%)	*Y*_TAG/xylose_ (%)	Residual xylose (g/l)	TAG (g/l)	*Y*_TAG/x_ (%)	Y_TAG/xylose_ (%)
WT	0.0	4.1 ± 0.2	66.1 ± 3.6	20.4 ± 0.9	0.0	3.9 ± 0.8	62.4 ± 13.5	20.7 ± 4.4
*PDC1*	0.0	4.3 ± 0.3	71.4 ± 6.2	21.7 ± 1.6	0.1 ± 0.2	4.7 ± 0.2	77.1 ± 3.2	25.2 ± 0.6^∗^
*PDC1, ALD6*	0.2 ± 0.32	4.0 ± 0.2	64.5 ± 3.6	20.2 ± 0.9	0.0	4.6 ± 0.5	75.3 ± 12.5	24.4 ± 2.5^∗^
*PDC1, ACS2*	0.0	4.0 ± 0.1	66.4 ± 1.9	20.1 ± 0.4	0.0	4.9 ± 0.1	84.1 ± 4.0	25.7 ± 0.5^∗^
*PDC1, PDAT*	0.0	3.7 ± 0.5	58.5 ± 4.0	18.7 ± 2.4	0.5 ± 0.5	4.6 ± 0.3	76.1 ± 5.3	24.9 ± 1.1^∗^
*PDC1, ALD6, ACS2*	0.0	4.3 ± 0.1	66.8 ± 3.4	21.5 ± 0.5	0.0	4.9 ± 0.1	79.0 ± 4.2	26.1 ± 0.6^∗^
*PDC1, ALD6, PDAT*	0.0	4.0 ± 0.3	62.4 ± 3.3	19.9 ± 1.7	2.8 ± 1.6	4.3 ± 0.6	81.9 ± 11.0	27.0 ± 1.2^∗^
*PDC1, ALD6, ACS2, PDAT*	0.0	3.7 ± 0.1	61.2 ± 0.6	18.3 ± 0.4	0.2 ± 0.2	4.8 ± 0.2	81.1 ± 1.0	25.7 ± 0.7^∗^

*Values which are significantly higher (*p* < 0.10) than the control are indicated with ^∗^.*

At C/N ratio 103, all strains, except *PDC1, ALD6*, and *PDAT* (2.8 g/l) had utilized most of the xylose within 94 h (<0.6 g/l, **Table 4**). TAG production was highest in the strains having *PDC1* and *ACS2* or *PDC1, ALD6*, and *ACS2* (4.9 ± 0.1 g/l TAG), other transformants producing 4.3–4.8 g/l and the control strain 3.9 ± 0.8 g/l. The *PDC1, ACS2* strain had the highest *Y*_TAG/x_ (84.1 ± 4.01%), whereas the other transformants had 75–82% *Y*_TAG/x_ (**Table 4**). The yield on xylose was higher (*p* < 0.10) with the transformants compared to the control strain (**Table 4**). Yield on xylose was the highest with the strain having *PDC1, ALD6*, and *PDAT* (27.0 ± 1.2), other strains having *Y*_TAG/S_ of 25–26% and the control only 20.7 ± 4.4% (**Table 4**).

### Bioreactor Cultivation in Minimal Medium at C/N Ratio 70

**FIGURE 5:**
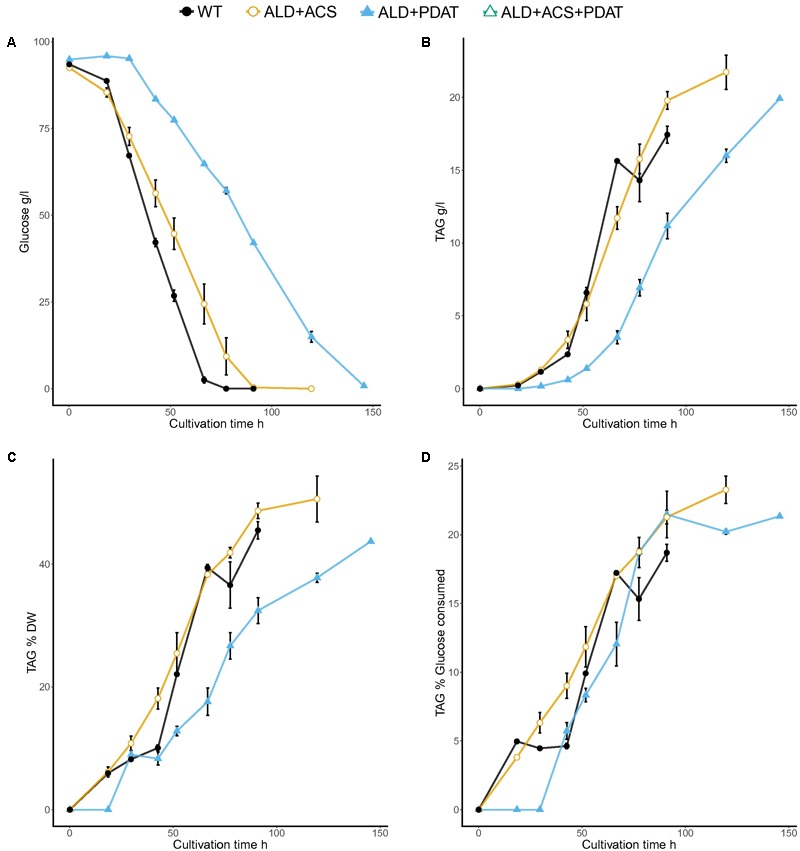
Glucose consumption **(A)** and TAG titer **(B)**, TAG yield on biomass **(C)**, and TAG yield on glucose **(D)** for *T. oleaginosus* grown in batch culture in medium with a C/N ratio of 70, at pH 4, 30°C, 1000 rpm. Error bars present ± SEM and if not visible are smaller than the symbol.

**FIGURE 6:**
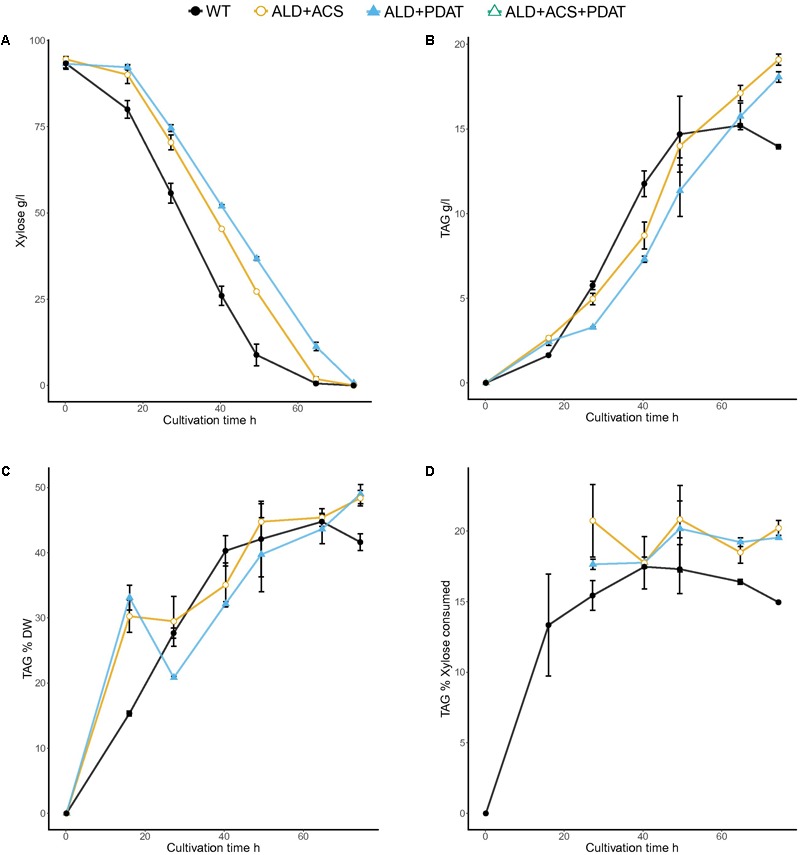
Xylose consumption **(A)** and TAG titer **(B)**, TAG yield on biomass **(C)**, and TAG yield on xylose **(D)** for *T. oleaginosus* grown in batch culture in medium with a C/N ratio of 70, at pH 4, 30°C, 1000 rpm. Error bars present ± SEM and if not visible are smaller than the symbol.

The transformants having *ALD6, ACS2*, and *PDAT* had high, but variable, yield of TAG on both glucose and xylose and the best transformant was selected for growth in bioreactor, along with the transformant having *ALD6* and *PDAT*, for which insufficient replicates had been available in the glucose flask cultures. The strains were grown in batch cultures, along with the control, with glucose (94 ± 0.4 g/l) or xylose (94 ± 0.6 g/l) as carbon source (**Figures 5, 6**). The control strain consumed all glucose within 78 h (1.4 g/l/h), whereas the transformant with *ALD6* and *PDAT* only within 91 h (1.2 g/l/h) and the strain having *ALD6, ACS2*, and *PDAT* within 146 h (0.8 g/l/h). Although the transformants were slower in glucose consumption than the control strain, they produced slightly (*p* < 0.06) more TAG (**Figure 5**). The *ALD6, ACS2*, and *PDAT* strain produced 20.0 ± 0.1 g/l TAG and the *ALD6* and *PDAT* strain 19.8 ± 0.9 g/l TAG, compared to 17.5 ± 0.8 g/l TAG in the control strain (**Figure 5**). There was no significant difference (*p* > 0.10) in the yield of TAG on biomass, although the transformant having *ALD6* and *PDAT* (48.7 ± 1.8%) contained slightly more TAG than the strain with *ALD6, ACS2*, and *PDAT* (43.7 ± 0.0%) or the control strain (45.5 ± 2.0%). However, both transformants had significantly (*p* < 0.05) higher yield of TAG on glucose (21.4 ± 0.1% and 21.3 ± 0.7%) than the control (18.7 ± 0.9%, **Figure 5**).

The bioreactor cultivations in minimal xylose medium were inoculated with high initial biomass, which resulted in fast xylose consumption (**Figure 6**). The control strain and transformant with *ALD6* and *PDAT* consumed almost all of the xylose within 65 h (1.7 and 1.8 g/l/h xylose consumption rate, respectively) and the transformant with *ALD6, ACS2*, and *PDAT* within 74 h (1.6 g/l/h). Both transformants produced significantly (*p* < 0.05) more TAG (18–19 g/l) at significantly (*p* < 0.05) higher yield (on biomass and on xylose) than the control strain (14 g/l, **Figure 6**). *Y*_TAG/x_ was 48.4 ± 1.7% and 49.0 ± 2.1% for the transformants, but only 44.8 ± 1.8% for the control. Y_TAG/S_ was 20.8 ± 1.8% and 20.2 ± 4.3% for the transformants, but 17.5 ± 0.1% for the control. Interestingly, high yield on substrate was observed within 40–49 h, when there was still substantial xylose (25–35 g/l residual xylose, **Figure 6**) in the culture broth and the effective C/N ratio would have been between 40 and 50.

## Discussion

We have established a PDH bypass in a xylose utilizing oleaginous yeast for the first time by expressing PDC, ALD, and ACS encoding genes. With the expression of the PDH bypass, our aim was to enhance yeast TAG production compared to the control strain of *T. oleaginosus*, which has only the native PDH pathway with ACL to generate cytosolic acetyl-CoA. A PDH bypass has previously only been expressed in the oleaginous yeast *Y. lipolytica*, which does not utilize xylose (Xu et al., 2016). The most common strategy for improving lipid production in yeast has been overexpression of the existing, endogenous pathways, with a strong focus on *Y. lipolytica* and *Rhodosporidium toruloides* (Probst et al., 2016; Adrio, 2017). However, Xu et al. (2016) reported that creating a PDH bypass in *Y. lipolytica* by expressing both *PDC* and *ALD* in a strain engineered to produce high amounts of lipid (*ACC1* and *DGA1* also overexpressed) increased the yield of lipid on glucose (*Y*_Lipid/S_) from 0.175 to 0.192 g/g, whereas overexpression of *ACL* did not affect the yield (*Y*_Lipid/S_ 0.173 g/g). The benefit of introducing the PDH bypass for TAG synthesis was in line with the 14.5% improvement we estimated with the genome-scale model simulations, in which the maximum attainable yields from glucose (i.e., without growth) were 0.252 g/g through the PDH route and 0.289 g/g through the PDH bypass route. When we introduced *PDC1* along with *ALD6* and *ACS2* encoding genes, a similar level of improvement was observed in the yield of TAG on glucose at low C/N ratio (from 0.197 to 0.216 g/g, **Table 3**) and better yields at high C/N ratio on glucose (0.233 g/g, **Table 3**) and on xylose (e.g., 0.27 compared to 0.21 g/g, **Table 4**). As observed by Xu et al. (2016), expression of the PDH bypass resulted in some improvement in TAG production at low C/N ratios, but the best TAG production was still observed with strong nitrogen limitation. The yield of TAG on xylose (0.27 g/g) in the engineered strain approached the theoretical maximum yield of 0.289 g/g.

The genome of our lipid production host, *T. oleaginosus*, was recently sequenced and assembled (Close and Ojumu, 2016). Two putative PDC genes have been found in the *T. oleaginosus* genome^3^, but these genes have very low identity to known, characterized *PDC* genes and do not necessarily encode PDCs. Even if *T. oleaginosus* has a functional endogenous PDC, its activity was low in lipid producing (aerobic) conditions: no ethanol was observed in any of the cultivations described here. Thus, we expected *PDC* expression to be required to complete the PDH bypass. Expression of *PDC1* alone appeared to enhance TAG titer and yields on both glucose and xylose (**Tables 2, 3**), although the difference was not conclusive because of the high variation observed between replicates. On both glucose and xylose, the best yield of TAG on substrate (*p* < 0.10) was observed in the strain which had *PDC1, ALD6*, and *PDAT*. In this combination there is a push (PDC) and pull (PDAT) effect, combined with NADPH production by ALD. The *S. cerevisiae PDC1* gene has also been expressed in *Y. lipolytica* together with the *S. cerevisiae ADH1* gene, but for the purpose of obtaining ethanol not lipid (Gatter et al., 2016).

The *T. oleaginosus* genome also contains one putative *ACS* gene, but no ALD encoding gene has been identified, leaving the PDH bypass pathway incomplete. Therefore, generation of the active PDH bypass required addition of *ALD6* from *S. cerevisiae*. *ALD6* is a cytosolic ALD which utilizes NADP^+^ as a cofactor in the reaction with acetaldehyde, producing acetate and NADPH (Saint-Prix et al., 2004). In theory, this NADPH would be beneficial in fatty acid synthesis, since in one cycle of fatty acid synthesis, two NADPHs are required. When NADP-dependent ALD is active, the flux through the PDH bypass will generate one NADPH for each cytosolic acetyl-CoA. The genome-scale metabolic model simulations showed that addition of ALD was beneficial when NADPH generation from the PPP was limited to the flux observed in wild type *Y. lipolytica* (Wasylenko et al., 2015). Although xylose enters metabolism through the non-oxidative part of the PPP, its metabolism does not generate NADPH, which may explain why the benefit to *Y*_TAG/x_ and *Y*_TAG/S_ of adding *ALD6* and *PDC1* to *T. oleaginosus* was greater on xylose (**Table 4**) than on glucose (**Table 3**). Addition of *ALD6* alone was not expected to improve lipid yields, but some benefit was again observed on xylose (**Figure 4**), but not glucose (**Figure 2**).

Although the genome sequence indicates that an *ACS* gene is present in *T. oleaginosus*, the *S. cerevisiae ACS2* was added along with *ALD6* to reduce glucose repression of acetyl-CoA formation. Acetate formed in the ALD reaction is converted into acetyl-CoA by acetyl-CoA synthase. *ACS2* is one of two *S. cerevisiae* ACS encoding genes. This isoform is not under glucose repression, as is the *ACS1* gene in *S. cerevisiae* (Van den Berg and Steensma, 1995). *ACS1* may also have post-translational regulation (Shiba et al., 2007). Interestingly, when *ACS2* was expressed alone in *T. oleaginosus*, without other heterologous genes involved in the PDH bypass, glucose and xylose consumption were faster than in the control strain (Supplementary Tables S1–S3). TAG titers at the end of cultivations and TAG yields during the cultivations were also higher in the *ACS2* expressing strain compared to the control, suggesting that other cytosolic routes to TAG synthesis were operating. Xu et al. (2016) demonstrated that overexpression of phosphoketolase from the PPP, which generates acetyl-phosphate, and phosphotransacetylase, converting acetyl-phosphate to acetyl-CoA, resulted in a high yield of lipid on glucose (0.225 g/g) in *Y. lipolytica*. Acetyl-phosphate can also be converted to acetate, with the generation of ATP, and an active phosphoketolase and acetate kinase may have been generating cytoplasmic acetate for *ACS2* to convert to acetyl-CoA.

To further enhance TAG production, we expressed the putative *PDAT* gene from *R. oryzae* in *T. oleaginosus*. PDAT, a phospholipid diacyltransferase, catalyzes the last step of TAG synthesis. Expression of *PDAT* or *DGAT* has been shown to enhance TAG production in *S. cerevisiae* and *Y. lipolytica* (Oelkers et al., 2002; Blazeck et al., 2014). The *T. oleaginosus* strain with only the *PDAT* gene from *R. oryzae* expressed consumed glucose slower than the control strain, but TAG titer and yields were better, especially at C/N ratio 103. The slow glucose consumption and good TAG yield were also seen in the strains expressing *PDAT* in combination with the PDH bypass genes. On glucose, TAG titer and yields were generally the highest in the strain with all three genes, implying that even partial expression of the PDH bypass together with *PDAT* expression enhanced TAG production yields. This was also observed in the bioreactor cultivations, which allowed higher TAG production than in the flasks, since higher substrate concentrations could be used. On glucose, transformants with either *ALD* and *PDAT* or *ALD, ACS*, and *PDAT* produced about 14% more TAG than the control strain, with a corresponding increase in the yield of TAG on glucose. On xylose, both transformants produced 19–26% more TAG, with the *Y*_TAG/xylose_ 15–19% higher, than the control strain. The higher yield on carbohydrate supported the hypothesis that it is possible to improve TAG yield by enhancing cytosolic acetyl-CoA production.

Lipid production has been extensively studied with modified *Y. lipolytica* strains (Shi and Zhao, 2017). With these strains, high lipid titers and yields have been achieved on glucose. For example, Qiao et al. (2017) reported 98.9 g/l lipid production with 27% *Y*_TAG/S_ and 66.8% *Y*_TAG/x_ in fed-batch cultivation with *Y. lipolytica* having *ACC1, DGA1, GapC* (*Clostridium acetobutylicum*) and *MCE2* (*Mucor circinelloides*) genes. However, *Y. lipolytica* cannot utilize xylose (Zhao et al., 2015). Even when genetically modified to express Scheffersomyces *stipitis XYL1* and *XYL2* genes, *Y. lipolytica* produced only 15 g/l lipids from 160 g/l xylose (Li and Alper, 2016). Addition of an extra copy of the endogenous *XKS1* gene improved production of lipid from xylose, but yields were still low (Ledesma-Amaro et al., 2016). Production of lipids from xylose has also been reported for *R. toruloides*, which is able to grow on xylose. The yield of lipid on either xylose or biomass was higher in both a wild type *R. toruloides* (11–16% *Y*_TAG/S_ and 36–45% *Y*_TAG/x_, Wiebe et al., 2012) and a genetically modified strain (*ACC1* and *DGA1*, 14% *Y*_TAG/S_ and 43.4% *Y*_TAG/x_, Zhang et al., 2016) than in the engineered *Y. lipolytica* (8% *Y*_TAG/S_ and 35% *Y*_TAG/x_, Ledesma-Amaro et al., 2016). While the wild type *T. oleaginosus* (15% *Y*_TAG/S_ and 42% *Y*_TAG/x_, **Figure 6**) had yields of TAG (which were 85% of total lipid) on xylose and biomass which were comparable to those of *R. toruloides* total lipids, addition of *ALD6* and *PDAT* (with or without *ACS2*) resulted in yields from xylose (20.8% *Y*_TAG/S_ and 48.4% *Y*_TAG/x_, **Figure 6**) which were comparable to those obtained from glucose and the highest so far reported for lipid or TAG production from xylose.

A “push and pull” strategy has been used successfully in oleaginous yeasts like *Y. lipolytica* and in *R. toruloides* to enhance their lipid production. In these cases, the genes encoding the first enzyme of fatty acid synthesis (ACC1) and the last enzyme of TAG synthesis (DGA1) were expressed (Tai and Stephanopoulos, 2013; Zhang et al., 2016; Qiao et al., 2017). Xu et al. (2016) demonstrated that pathways which increase cytoplasmic acetyl-CoA can be modified to further enhance lipid production. Here the provision of cytoplasmic acetyl CoA via the PDH bypass (and other reactions associated with the partial pathway) provided the “push,” while PDAT provided the “pull” to enhance lipid production. The PDH bypass was also expected to reduce the dependence of lipid production on nitrogen limitation (Xu et al., 2016), but improvements in lipid production in *T. oleaginosus* were primarily seen in cultures with high C/N ratios. Our results show that providing a route to cytoplasmic acetyl CoA, e.g., via the PDH bypass, was particularly useful when xylose was the substrate for lipid production, with the main benefit being the increased yield of TAG on substrate.

## Author Contributions

KK was responsible for all strain engineering and flask cultivations. MW for was responsible for bioreactor cultivations. PJ and SC for metabolic modeling. KK, LR, MP, and MW conceived the project and contributed to its design. KK, MW, and PJ drafted the manuscript, which all authors reviewed and edited.

## Conflict of Interest Statement

The authors are employees of VTT Technical Research Centre of Finland Ltd. and declare that part of the funding for this research was received from Neste, a company with interest in renewable diesel production.

## Footnotes

1 https://www.ncbi.nlm.nih.gov/pmc/articles/PMC5516929/

2 https://www.ncbi.nlm.nih.gov/pubmed/23927696

3 https://genome.jgi.doe.gov/Crycu1/Crycu1.home.html

## References

[B1] Adrio J. L. (2017). Oleaginous Yeasts: promising platforms for the production of oleochemicals and biofuels. *Biotech. Bioeng.* 114 1915–1920. 10.1002/bit.26337 28498495

[B2] Blazeck J. Hill A. Liu L. Knight R. Miller J. Pan A. (2014). Harnessing *Yarrowia lipolytica* lipogenesis to create a platform for lipid and biofuel production. *Nat. Commun.* 5:3131. 10.1038/ncomms4131 24445655

[B3] Burgard A. P. Vaidyaraman S. Maranas C. D. (2001). Minimal reaction sets for *Escherichia coli* metabolism under different growth requirements and uptake environments. *Biotechnol. Prog.* 17 791–797. 10.1021/bp0100880 11587566

[B4] Close D. Ojumu J. (2016). Draft genome sequence of the oleaginous yeast *Cryptococcus curvatus* ATCC 20509. *Genome Announc.* 4:e01235-16. 10.1128/genomeA.01235-16 27811111 PMC5095481

[B5] Flikweert M. T. Van der Zanden L. Janssen W. M. Steensma H. Y. Van Dijken J. P. Pronk J. T. (1996). Pyruvate decarboxylase: an indispensable enzyme for growth of *Saccharomyces cerevisiae* on glucose. *Yeast* 12 247–257. 10.1002/(SICI)1097-0061(19960315)12:3<247::AID-YEA911>3.0.CO;2-I 8904337

[B6] Folch J. Lees M. Stanley G. H. S. (1957). A simple method for the isolation and purification of total lipids from animal tissues. *J. Biol. Chem.* 271 28953–28959. 13428781

[B7] Gatter M. Ottlik S. Kövesi Z. Bauer B. Matthäus F. Barth G. (2016). Three alcohol dehydrogenase genes and one acetyl-CoA synthetase gene are responsible for ethanol utilization in *Yarrowia lipolytica*. *Fungal Gen. Biol.* 95 30–38. 10.1016/j.fgb.2016.07.012 27486067

[B8] Ledesma-Amaro R. Lazar Z. Rakicka M. Guo Z. Fouchard F. Crutz-LeCoq A.-M. (2016). Metabolic engineering of *Yarrowia lipolytica* to produce chemicals and fuels from xylose. *Metab. Eng.* 38 115–124. 10.1016/j.ymben.2016.07.001 27396355

[B9] Li H. Alper H. S. (2016). Enabling xylose utilization in *Yarrowia lipolytica* for lipid production. *Biotechnol. J.* 11 1230–1240. 10.1002/biot.201600210 27367454

[B10] Mach R. L. Schindler M. Kubicek C. P. (1994). Transformation of *Trichoderma reesei* based on hygromycin B resistance using homologous expression signals. *Curr. Genet.* 25 567–570. 10.1007/BF00351679 8082210

[B11] Mahadevan R. Schilling C. (2003). The effects of alternate optimal solutions in constraint-based genome-scale metabolic models. *Metab. Eng.* 5 264–276. 10.1016/j.ymben.2003.09.002 14642354

[B12] Mueller P. R. Wold B. (1989). In vivo footprinting of a muscle specific enhancer by ligation mediated PCR. *Science* 246 780–786. 10.1126/science.2814500 2814500

[B13] Nakazawa N. Hashimoto H. Harashima S. Oshima Y. (1993). Use of the *PDR4* gene as a dominant selective marker in combination with cerulenin for prototrophic strains in *Saccharomyces cerevisiae*. *J Ferment. Bioeng.* 76 60–63. 10.1016/0922-338X(93)90054-C

[B14] Oelkers P. Cromley D. Padamsee M. Billheimer J. T. Sturley S. L. (2002). The *DGA1* gene determines a second triglyceride synthetic pathway in yeast. *J. Biol. Chem.* 277 8877–8881. 10.1074/jbc.M111646200 11751875

[B15] Probst K. V. Schulte L. R. Durrett T. P. Rezac M. E. Praveen V. V. (2016). Oleaginous yeast: a value-added platform for renewable oils. *Crit. Rev. Biotechnol.* 36 942–955. 10.3109/07388551.2015.1064855 26180999

[B16] Pronk J. T. Steensma H. Y. Van Dijken J. P. (1996). Pyruvate metabolism in *Saccharomyces cerevisiae*. *Yeast* 12 1607–1633. 10.1002/(SICI)1097-0061(199612)12:16<1607::AID-YEA70>3.0.CO;2-4 9123965

[B17] Qiao K. Wasylenko T. M. Zhou K. Xu P. Stephanopoulos G. (2017). Lipid production in *Yarrowia lipolytica* is maximized by engineering cytosolic redox metabolism. *Nat. Biotech.* 35 173–177. 10.1038/nbt.3763 28092657

[B18] Ratledge C. Wynn J. P. (2002). The biochemistry and molecular biology of lipid accumulation in oleaginous microorganisms. *Adv. Appl. Microbiol.* 51 1–51. 10.1016/S0065-2164(02)51000-5 12236054

[B19] Saint-Prix F. Bonquist L. Dequin S. (2004). Functional analysis of the *ALD* gene family of *Saccharomyces cerevisiae* during anaerobic growth on glucose: the NADP+ -dependent Ald6p and Ald5p isoforms play a major role in acetate formation. *Microbiology* 150 2209–2220. 10.1099/mic.0.26999-0 15256563

[B20] Shi S. Zhao H. (2017). Metabolic engineering of oleaginous yeasts for production of fuels and chemicals. *Front. Microbiol.* 8:2185 10.3389/fmicb.2017.02185 PMC5682390 29167664

[B21] Shiba Y. Paradise E. M. Kirby J. Ro D.-K. Keasling J. D. (2007). Engineering of the pyruvate dehydrogenase bypass in *Saccharomyces cerevisiae* for high-level production of isoprenoids. *Metab. Eng.* 9 160–168. 10.1016/j.ymben.2006.10.005 17196416

[B22] Sorger D. Daum G. (2003). Triacylglycerol biosynthesis in yeast. *Appl. Microbiol. Biotechnol.* 61 289–299. 10.1007/s00253-002-1212-4 12743757

[B23] Tai M. Stephanopoulos G. (2013). Engineering the push and pull of lipid biosynthesis in oleaginous yeast *Yarrowia lipolytica* for biofuel production. *Metab. Eng.* 15 1–9. 10.1016/j.ymben.2012.08.007 23026119

[B24] Tehlivets O. Scheuringer K. Kohlwein S. D. (1997). Fatty acid synthesis and elongation in yeast. *Biochim. Biophys. Acta* 1771 255–270. 10.1016/j.bbalip.2006.07.004 16950653

[B25] Van den Berg M. A. Steensma H. Y. (1995). *ACS2*, a *Saccharomyces cerevisiae* gene encoding acetyl-coenzyme A synthetase essential for growth on glucose. *Eur. J. Biochem.* 231 704–713. 10.1111/j.1432-1033.1995.tb20751.x 7649171

[B26] van Rossum H. M. Kozak B. U. Pronk J. T. van Maris A. J. A. (2016). Engineering cytosolic acetyl-coenzyme A supply in *Saccharomyces cerevisiae*: pathway stoichiometry, free-energy conservation and redox-cofactor balancing. *Metab. Eng.* 36 99–115. 10.1016/j.ymben.2016.03.006 27016336

[B27] Wasylenko T. M. Ahn W. S. Stephanopoulos G. (2015). The oxidative pentose phosphate pathway is the primary source of NADPH for lipid overproduction from glucose in *Yarrowia lipolytica*. *Metab. Eng.* 30 27–39. 10.1016/j.ymben.2015.02.007 25747307

[B28] Wiebe M. G. Koivuranta K. Penttilä M. Ruohonen L. (2012). Lipid production in batch and fed-batch cultures of *Rhodosporidium toruloides* from 5 and 6 carbon carbohydrates. *BMC Biotech.* 12:26. 10.1186/1472-6750-12-26 22646156 PMC3499381

[B29] Xu P. Qiao K. Ahn W. S. Stephanopoulos G. (2016). Engineering *Yarrowia lipolytica* as a platform for synthesis of drop-in transportation fuels and oleochemicals. *Proc. Natl. Acad. Sci. U.S.A.* 113 10848–10853. 10.1073/pnas.1607295113 27621436 PMC5047176

[B30] Zhang S. Skerker J. M. Rutter C. D. Maurer M. J. Arkin A. P. Rao C. V. (2016). Engineering *Rhodosporidium toruloides* for increased lipid production. *Biotech. Bioeng.* 113 1056–1066. 10.1002/bit.25864 26479039

[B31] Zhao C. Gu D. Nambou K. Wei L. Chen J. Imanaka T. (2015). Metabolome analysis and pathway abundance profiling of *Yarrowia lipolytica* cultivated on different carbon sources. *J. Biotech.* 206 42–51. 10.1016/j.jbiotec.2015.04.005 25912211

