# Supplementary material for: Enhanced Triacylglycerol Production With Genetically Modified Trichosporon oleaginosus
Source: Front Microbiol. 2018 Jun 21;9:1337. doi: 10.3389/fmicb.2018.01337 (PMC6021488; doi:10.3389/fmicb.2018.01337)
Supplement: Supplementary file 1 [file Table_1.PDF]

Supplementary Material

Enhanced Triacylglycerol Production with Genetically Modified *Trichosporon oleaginosus*

Kari Koivuranta, Sandra Castillo, Paula Jouhten, Laura Ruohonen †, Merja Penttilä, and Marilyn G. Wiebe*

*** Correspondence:** Marilyn Wiebe: Marilyn.wiebe@VTT.fi

Table S1. Residual glucose concentration and TAG production (titer and yields $Y_{TAG/x}$ and $Y_{TAG/glucose}$) from glucose at C/N ratio 65 in flask cultivation. Values are average ± SEM. Values at 68 h which are significantly higher than the control are indicated with * ($p < 0.10$) or † ($p < 0.05$).

Time (h)	0	20	25	44	49	68
Strain	Glucose (g l^{-1})					
Control	18.0	10.5 ± 0.5	8.1 ± 0.4	0	0	0
ALD6	18.0	10.6 ± 0.4	8.6 ± 0.4	1.0 ± 0.4	0.1 ± 0.1	0
ALD6, ACS2	18.0	11.3 ±0.4	9.2 ± 0.5	1.3 ± 0.5	0.2 ± 0.1	0
ACS2	18.0	11.4 ± 0.6	9.3 ± 0.8	1.4 ±1.0	0.7 ± 0.7	0
PDAT	18.0	12.6 ±1.2	10.6 ± 1.8	5.0 ± 1.9	3.7 ± 2.0	1.1 ± 1.1
ALD6, PDAT	18.0	13.7	12.2	5.4	3.9	0
ALD6, ACS2, PDAT	18.0	13.8 ± 0.2	12.4 ± 0.4	6.4 ± 0.7	5.0 ± 0.8	0.8 ± 0.5
	TAG (g l^{-1})					
Control	0	1.0 ± 0.1		3.0 ± 0.1		3.4 ± 0.0
ALD6	0	1.1 ± 0.1		2.9 ± 0.1		3.1 ± 0.1
ALD6, ACS2	0	1.0 ± 0.1		2.9 ± 0.1		3.0 ± 0.1
ACS2	0	1.0 ± 0.1		2.9 ± 0.2		3.6 ± 0.1†
PDAT	0	0.7 ± 0.3		2.2 ± 0.2		3.1 ± 0.0
ALD6, PDAT	0	0.7		2.4		3.0
ALD6, ACS2, PDAT	0	0.6 ± 0.0		2.5 ± 0.1		3.7 ± 0.3
	TAG yield ($Y_{TAG/x}$, g TAG per g biomass)					
Control	0	21.7 ± 2.3		40.0 ± 1.0		47.5 ± 1.1
ALD6	0	22.0 ± 2.6		40.4 ± 1.4		39.5 ± 2.1
ALD6, ACS2	0	23.0 ± 1.9		40.8 ± 2.3		43.0 ± 1.8
ACS2	0	20.3 ± 1.4		38.5 ± 0.6		52.6 ± 1.5†
PDAT	0	16.4 ± 2.7		38.8 ± 4.4		46.0 ± 5.7
ALD6, PDAT	0	21.1		42.8		40.4
ALD6, ACS2, PDAT	0	14.2 ± 1.4		43.0 ± 4.2		58.8 ± 3.2*
	TAG yield ($Y_{TAG/glucose}$, g TAG per g glucose consumed)					
Control	0	10.3 ± 0.2		16.6 ± 0.7		19.1 ± 0.2
ALD6	0	11.2 ± 0.8		17.3 ± 0.4		17.0 ± 0.8
ALD6, ACS2	0	11.7 ± 0.8		17.2 ± 0.6		16.7 ± 0.7
ACS2	0	11.7 ± 0.2		17.6 ± 0.2		20.1 ± 0.4†
PDAT	0	8.8 ± 1.6		16.9 ± 0.7		18.2 ± 1.4
ALD6, PDAT	0	12.7		18.6		16.8
ALD6, ACS2, PDAT	0	8.0 ± 0.2		17.4 ± 0.6		20.5 ± 1.9

Table S2. Residual glucose concentration and TAG production (titer and yields $Y_{TAG/x}$ and $Y_{TAG/glucose}$) from glucose at C/N ratio 103 in flask cultivation. Values are average ± SEM. Values at 83.5 h which are significantly higher than the control are indicated with * ($p < 0.10$) or † ($p < 0.05$).

Time (h)	0	17.5	22.5	35	40	59	64	83.5
Strain	Glucose (g l^{-1})							
Control	18.2	13.0 ± 0.4	11.9 ± 0.4	5.9 ± 0.0	5.7 ± 0.8	0.1 ± 0.1	0	0
ALD6	18.2	13.8 ± 0.3	12.2 ± 0.5	6.8 ± 0.3	6.1 ± 0.3	1.1 ± 0.4	0.2 ± 0.2	0
ALD6, ACS2	18.2	13.7 ± 0.2	12.3 ± 0.3	6.8 ± 0.3	6.2 ± 0.3	1.4 ± 0.4	0.6 ± 0.4	0
ACS2	18.2	13.6 ± 0.2	12.0 ± 0.4	6.3 ± 0.2	5.5 ± 0.2	0.2 ± 0.1	0	0
PDAT	18.2	13.7 ± 0.2	12.3 ± 0.3	6.5 ± 0.3	5.6 ± 0.3	0.9 ± 0.7	0.4 ± 0.4	0
ALD6, PDAT	18.2	14.2	12.8	7.7	7.2	3.4	2.6	0
ALD6, ACS2, PDAT	18.2	13.9 ± 0.3	13.0 ± 0.1	7.8 ± 0.0	6.8 ± 0.0	1.9 ± 0.0	0.9 ± 0.0	0
	TAG (g l^{-1})							
Control	0		0.6 ± 0.1	2.0 ± 0.0		3.7 ± 0.1		4.0 ± 0.1
ALD6	0		0.7 ± 0.1	1.9 ± 0.1		3.6 ± 0.1		4.0 ± 0.1
ALD6, ACS2	0		0.5 ± 0.1	2.0 ± 0.1		3.5 ± 0.1		4.1 ± 0.1
ACS2	0		0.6 ± 0.1	2.0 ± 0.1		3.5 ± 0.1		4.1 ± 0.1
PDAT	0		0.7 ± 0.1	2.1 ± 0.2		3.7 ± 0.2		4.2 ± 0.1†
ALD6, PDAT	0		0.6	2.22		3.4		3.7
ALD6, ACS2, PDAT	0		0.7 ± 0.1	1.6 ± 0.1		2.6 ± 0.6		4.3 ± 0.1†
	TAG yield ($Y_{TAG/x}$, g TAG per g biomass)							
Control	0		19.4 ± 5.0	36.4 ± 3.6		56.5 ± 1.9		63.5 ± 3.4
ALD6	0		24.6 ± 4.7	39.9 ± 4.2		54.9 ± 1.6		65.7 ± 3.5
ALD6, ACS2	0		16.8 ± 4.0	37.4 ± 3.2		54.4 ± 3.2		56.1 ± 2.2
ACS2	0		28.9 ± 6.5	47.2 ± 3.7		54.8 ± 2.1		60.7 ± 1.6
PDAT	0		22.8 ± 5.4	46.2 ± 5.0		63.5 ± 3.1		67.6 ± 5.3
ALD6, PDAT	0		28.1	49.4		67.8		62.4
ALD6, ACS2, PDAT	0		17.5 ± 0.6	37.5 ± 4.3		43.3 ± 6.3		66.0 ± 6.3
	TAG yield ($Y_{TAG/glucose}$, g TAG per g glucose consumed)							
Control	0		9.9 ± 2.4	16.1 ± 0.1		20.2 ± 0.7		21.8 ± 0.3
ALD6	0		12.8 ± 2.1	16.7 ± 1.1		19.8 ± 0.4		22.1 ± 0.3
ALD6, ACS2	0		9.1 ± 1.5	17.0 ± 1.1		19.6 ± 0.3		22.4 ± 0.4
ACS2	0		10.7 ± 1.7	16.9 ± 0.1		19.2 ± 0.3		22.7 ± 0.3
PDAT	0		11.1 ± 1.4	17.7 ± 0.9		21.0 ± 0.5		23.0 ± 0.5†
ALD6, PDAT	0		10.5	21.1		21.8		20.6
ALD6, ACS2, PDAT	0		7.6 ± 1.2	15.0 ± 0.9		15.2 ± 3.5		23.4 ± 0.5†

Table S3. Residual xylose concentration and TAG production (titer and yields $Y_{TAG/x}$ and $Y_{TAG/xylose}$) from xylose at C/N ratio 103 in flask cultivation. Values are average ± SEM. Values at 70 h which are significantly higher than the control are indicated with * ($p < 0.10$) or † ($p < 0.05$).

Time (h)	0	21	45	51.5	69.5
Strain	Xylose (g l^{-1})				
Control	19.2	15.9 ± 0.1	11.2 ± 0.3	10.0 ± 0.3	5.0 ± 0.4
ALD6	19.2	14.9 ± 0.3	9.2 ± 0.4	7.7 ± 0.7	3.2 ± 0.5
ALD6, ACS2	19.2	15.3 ± 0.2	10.0 ± 0.4	8.4 ± 0.7	3.9 ± 0.6
ACS2	19.2	15.0 ± 0.4	9.3 ± 0.5	7.2 ± 0.8	2.7 ± 0.5
PDAT	19.2	14.4 ± 0.3	9.4 ± 0.1	8.4 ± 0.1	3.5 ± 0.3
ALD6, PDAT	19.2	16.3 ± 0.1	12.3 ± 0.1	11.4 ± 0.1	8.3 ± 0.7
ALD6, ACS2, PDAT	19.2	15.6 ± 0.1	10.5 ± 0.2	9.4 ± 0.2	4.7 ± 0.3
	TAG (g l^{-1})				
Control	0	0.2 ± 0.0		1.2 ± 0.1	2.9 ± 0.2
ALD6	0	0.3 ± 0.1		1.7 ± 0.1	4.0 ± 0.1†
ALD6, ACS2	0	0.3 ± 0.0		1.6 ± 0.1	3.5 ± 0.2†
ACS2	0	0.3 ± 0.0		1.8 ± 0.1	3.9 ± 0.1†
PDAT	0	0.3 ± 0.0		1.8 ± 0.4	3.8 ± 0.3†
ALD6, PDAT	0	0.1 ± 0.0		1.7 ± 0.1	2.7 ± 0.1
ALD6, ACS2, PDAT	0	0.2 ± 0.0		2.1 ± 0.0	3.4 ± 0.2*
	TAG yield ($Y_{TAG/x}$, g TAG per g biomass)				
Control	0	11.8 ± 0.4		27.1 ± 2.4	63.8 ± 3.6
ALD6	0	13.9 ± 1.7		36.3 ± 1.3	75.9 ± 1.1*
ALD6, ACS2	0	12.1 ± 1.8		39.9 ± 2.5	64.8 ± 2.5
ACS2	0	13.3 ± 1.8		42.9 ± 2.1	71.1 ± 5.7
PDAT	0	13.4 ± 1.1		43.3 ± 8.3	72.7 ± 5.3
ALD6, PDAT	0	7.6 ± 1.1		54.8 ± 5.2	73.0 ± 6.6
ALD6, ACS2, PDAT	0	13.3 ± 1.3		56.7 ± 1.1	67.5 ± 3.4
	TAG yield ($Y_{TAG/xylose}$, g TAG per g xylose consumed)				
Control	0	4.5 ± 0.1		13.2 ± 0.7	20.3 ± 0.5
ALD6	0	7.4 ± 1.6		14.7 ± 0.6	24.9 ± 0.6†
ALD6, ACS2	0	6.5 ± 0.8		14.8 ± 0.5	22.8 ± 0.4
ACS2	0	6.3 ± 0.1		15.3 ± 0.4	23.7 ± 0.1†
PDAT	0	6.3 ± 0.1		17.0 ± 3.4	24.4 ± 1.3†
ALD6, PDAT	0	5.1 ± 1.3		22.0 ± 1.8	24.4 ± 1.1†
ALD6, ACS2, PDAT	0	6.8 ± 0.6		21.6 ± 0.4	23.3 ± 0.9†

Table S4. Primers used throughout this study.

Primer name	Sequence (5’ - 3’)	Usage
PCR linker I	GCGGTGACCCGGGAGATCTGAATTC	Ligation-mediated PCR amplification
PCR linker II	GAATTCAGATCT	Ligation-mediated PCR amplification
Yeast TEF1	TACAAGTGYGGTGGTATYGACAAG	Cloning of TEF1 gene
Yeast TEF4	TCWACGGAYTTGACTTCAGTGGT	Cloning of TEF1 gene
CC_TEF2	CACGCTCACGCTCGGCC	TEF1 promoter
CC_TEF1	CCGAGGTCGGCGGCC	TEF1 promoter, nested
CC_TEF6	GGCAGGCGCAAAGCTGGAC	TEF1 promoter
CC_TEF5	GCAGTCACTGTCATTGTCGCACTACC	TEF1 promoter, nested
CC_TEF10_SacII	TCCCCGCGGGGATCCATCACGCCTGCCCGTCC	Cloning of TEF1 promoter
CC_TEF11_XbaI	GCTCTAGAGCCTGCAGGTTTTTATAGGTTCTGCGAATGGTTAGTACG	Cloning of TEF1 promoter
CC_TEF3	CCTCCAGGACGTCTACAAGATCGGC	TEF1 terminator
CC_TEFE4	CCCGTCGGCCGTGTCG	TEF1 terminator, nested
CC_TEF7_XmaI	TCCCCCCGGGCCTGCAGGTTGTAGAGCCCTCGGTTCTG	Cloning of TEF1 terminator
CC_TEF8_EcoRI	GGAATTCCGGAGGCTTGTCATCATACGAGAC	Cloning of TEF1 terminator
Yeast TPI5	GGTAACTKKAAGATGAACGGCTC	Cloning of TPI1 gene
Yeast TPI8	GCKCCRCCGACRAGGAAWCCRTC	Cloning of TPI1 gene
CC_TPI2	GGGAAGTTGGCGCTGTGGAC	TPI1 promoter
CC_TPI1	CGCTGAGCTTGGCGTCG	TPI1 promoter, nested
CC_TPI7_BamHI	CGGGATCCCGGAATTCCTGACCACCCGCG	Cloning of TPI1 promoter
CC_TPI9_SbfI	TGTGTGCCTGCAGGCTTGGATATGCTGTTTTAGGTTTGG	Cloning of TPI1 promoter
CC_TPI4	AAGCGCGTCTCGCAGAAGG	TPI1 terminator
CC_TPI3	ACGGCGGCTCCGTCAAC	TPI1 terminator, nested
CC_TPI5_XbaI	GCTCTAGAGCCTGCAGGGATGAGGCGTGGCATAGG	Cloning of TPI1 terminator
CC_TPI6_BamHI	CGGGATCCCGCAGCTGACGACAGGCT	Cloning of TPI1 terminator
Yeast ENO5	CCCGTCACCTCYCAGAAGGAGATTG	Cloning of ENO1 gene
Yeast ENO10	CGGTCTCACCGGATCGGTG-	Cloning of ENO1 gene
CC_ENO2	TTGGCAGCGGCCTCGG	ENO1 promoter

**Primer name**	**Sequence (5’ - 3’)**	**Usage**
CC_ENO1	CCAAGGATGGCGTTGGCG	ENO1 promoter, nested
CC_ENO5	CGTGCTCCTGCCCAGGAGG	ENO1 promoter
CC_ENO6	CGATGCTCTCGGCAGTTGCG	ENO1 promoter, nested
CC_ENO9_EcoRI	CGGAATTCTGTCTGTACGAGTCTGTACAC	Cloning of ENO1 promoter
CC_ENO10_EcoRI	GGAATTCCTGCAGGTTTGAGGTGAGGTTGTTGTTTTGG	Cloning of ENO1 promoter
CC_ENO4	GGCGTGCAACGCCCTCC	ENO1 terminator
CC_ENO3	CCATCCAGGCGTGGGTACTGA	ENO1 terminator, nested
CC_ENO7_HindIII	CCCAAGCTTCCTGCAGGGTGCGCGTAGTGCGC	Cloning of ENO1 terminator
CC_ENO8_HindIII	CCCAAGCTTGGGACGCCGAGGAGCATCTC	Cloning of ENO1 terminator
CC_GPD3	CGTCGGTCTTTGACGCCAAGG	GPD1 terminator
CC_GPD4	CGTGGTACGACAACGAGTACGGC	GPD1 terminator, nested
CC_GPD6CC_GPD6_XbaI	GCTCTAGAGCCTGCAGGATCCCTTCGAGGATGTAGTTAGGTTG	Cloning of GPD1 terminator
CC_GPD7_BamHI	CGGGATCCCGTGGAGGTGTCTGTGATGACGA	Cloning of GPD1 terminator
Hph 5_SpeI	GGACTAGTCCTGCAGGATGAAAAAGCCTGAACTCACCG	Cloning of hygromycin resistance gene
Hph 3_SpeI	GGACTAGTCCTGCAGGCTATTCCTTTGCCCTCGGACG	Cloning of hygromycin resistance gene
Kan 5_SpeI	GGACTAGTCCTGCAGGATGAGCCATATTCAACGGG	Cloning of G418 resistance gene
Kan 3_SpeI	GGACTAGTCCTGCAGGTTAGAAAAACTCATCGAGCATCA	Cloning of G418 resistance gene
CERR 5_SbfI	ACACACCCTGCAGGATGAGTGTGTCTACCGCCAAGAGG-	Cloning of cerulenin resistance gene
CERR 3_SbfI	GTGTGTCCTGCAGGTTAATTTGCGGCCGGTACCG	Cloning of cerulenin resistance gene
